# THE RADIOTHERAPY USE BY OLDER CANCER PATIENTS IN PORTUGAL—2018 NATIONAL CANCER REGISTRY DATA

**DOI:** 10.1093/geroni/igad104.2430

**Published:** 2023-12-21

**Authors:** Edna Darlene Rodrigues, Maria José Bento, Rita Calisto, Jessica Rodrigues, Paulo Almeida, Escarlata Lopez, Laetitia Teixeira

**Affiliations:** School of Medicine and Biomedical Sciences, University of Porto, Porto, Porto, Portugal; Portuguese Oncology Institute of Porto (IPO Porto), Porto, Porto, Portugal; Portuguese Oncology Institute of Porto (IPO Porto), Porto, Porto, Portugal; Portuguese Oncology Institute of Porto (IPO Porto), Porto, Porto, Portugal; Centro Hospitalar Universitário São João, Porto, Porto, Portugal; GenesisCare Spain, Madrid, Madrid, Spain; School of Medicine and Biomedical Sciences, University of Porto, Porto, Porto, Portugal

## Abstract

In Portugal, cancer is the second leading cause of death, accounting for 28544 in 2018, around 25% of all deaths. Radiotherapy (RT) will be required by around half of the cancer patients. Older patients should be treated with ultrahipofractionation and highly precise/accurate technology to reduce the treatment sessions and to avoid side effects which are important for their quality of life. Nowadays, there is a global lack of RT infrastructure/investment that compromises cancer outcomes. The aim of this study was to evaluate the RT coverage by older patients in Portugal. The authors examined the 2018 national cancer registry data regarding the RT use stratified by cancer site and age groups. A total of 14448 cancer registries regarding patients aged 70 y/o or over were included. We calculated the optimal use through the application of the optimal utilisation percentage of RT for each cancer site, published by European SocieTy for Radiotherapy and Oncology - Health Economics in Radiation Oncology (ESTRO-HERO) project. The ratio between optimal use and real RT use was 56%, 51%, 41%, and 36% for the patients aged 70-74, 75-79, 80-84, and 85+, respectively. We should interpret these results considering the complex older patients’ setting of multi-morbidity and geriatric syndromes. Furthermore, radiation oncologists face a lack of evidence that supports optimal treatment decisions for older patients. There is an urgent need for older adults’ inclusive clinical trials, measures to reduce ageism, improve access, and provide the cornerstone of quality cancer care for older adults - the geriatric assessment.

